# Effect of pH on the Hydrolytic Kinetics of Gamma-Glutamyl Transferase from *Bacillus subtilis*

**DOI:** 10.1155/2014/216270

**Published:** 2014-02-24

**Authors:** Sharath Balakrishna, Asmita Prabhune

**Affiliations:** Division of Biochemical Sciences, Room No. 1846, National Chemical Laboratory, Dr. Homi Bhabha Road, Pune 411008, India

## Abstract

The effect of pH on the steady state kinetics of gamma-glutamyl transferase (GGT) from *Bacillus subtilis* was examined using glutamyl-(3-carboxyl)-4-nitroanilide as the chromogenic reporter substrate. The enzyme was active in the pH range 7.0–11.0 with the optimum activity at pH 11.0. We noticed a pH dependent transformation in the nature of substrate consumption kinetics. The substrate saturation curves were hyperbolic in the pH range 7.0–9.0 but changed into sigmoid form at pH 10.0 and 11.0. Hill's coefficients were >1. We also analysed the effect of pH on the structure of the enzyme. The circular dichroism spectra of the enzyme sample at pH 9.0 and 11.0 were coincidental in both far and near UV regions indicating conservation of the secondary and tertiary structures, respectively. The molecular weight of the enzyme sample was the same in both pH 7.0 and 11.0 indicating conservation of the quaternary structure. These results show that the kinetic transformation does not involve significant conformational changes. Cooperative binding of multiple substrate molecules may not be the basis for the sigmoid kinetics as only one substrate binding site has been noticed in the reported crystal structures of *B. subtilis* GGT.

## 1. Introduction

Gamma-glutamyl transferases (GGT) (E.C.2.2.3.2) are a family of highly conserved enzymes occurring in archaebacteria, eubacteria, fungi, protozoa, nematodes, plants, and mammals [1]. They catalyse the exogenous removal of the terminal *γ*-glutamyl moiety from amides or peptides and its subsequent transfer to water (hydrolysis) or an acceptor amine (transpeptidation) or a second substrate molecule (autotransfer). The acceptor amine can be a peptide or an amino acid with a free amino group. The three reactions are represented below:

(1) γGluCONHR+H2O⟷Glu+RNH2(Hydrolysis)γGluCONHR1+R2CONH2⟷γGluCOR2+R1NH2(Transpeptidation)2γGluCONHR⟷γGlu-GluCONHR+RNH2(Autotranspeptidation)

Mammalian GGTs are involved in xenobiotic detoxification [2] and homeostasis of glutathione [3]. Human GGT plays a role in pathologies like metastasis [4] and drug resistance of malignant cells [5], cardiovascular diseases [6], inflammation [7], and diabetes [8] and neurodegenerative diseases [9]. Plant GGTs are speculated to participate in the synthesis of flavour compounds [10]. There are indications that *Bacillus subtilis* GGT catalyses the degradation of capsular *γ*-polyglutamic acid [11] while its homologue in *Bacillus anthracis* (capD) mediates covalent anchorage of capsular *γ*-poly-D-glutamic acid to the cell wall [12]. In *Helicobacter pylori* and *Neisseria meningitidis*, GGT helps the bacterium to colonize the intestinal epithelium [13] and the brain [14], respectively. So far, no unifying function has been found to explain the strong conservation across the phylogenetic tree. We are interested in the structure-function relationships of GGT. We have crystallised *B. subtilis* GGT and determined its X-ray crystallographic structure [15]. In the present study, we explore the effect of pH on the steady state kinetics of *B. subtilis* GGT and show its unique properties.

## 2. Materials and Methods

### 2.1. Chemicals

Glutamyl-(3-carboxyl)-4-nitroaniline ammonium salt was obtained from Fluka (Switzerland). All other reagents used were from Sigma Chemicals (USA).

### 2.2. Cloning and Expression of *B. subtilis* GGT

The GGT ORF was amplified from the genomic DNA of *B. subtilis* strain with the primers 5′ACG CGG CCA TGG TAA AGC CGC CCA AAA GCT3′ (forward) and 3′GTT TTT CTC GAG TTT ACG TTT TAA ATT GCC GAT5′ (reverse). The primers append 5′ and 3′ termini of the amplicon with flanks bearing recognition sites for the restriction enzymes *NcoI* and *XhoI*, respectively. The amplicon was produced by 25 cycles of Taq DNA polymerase catalysed polymerase chain reaction. Each thermal cycle comprised of 30 s denaturation at 94°C, 30 s annealing at 50°C and 90 s extension at 72°C. Both amplicon and pET26b (Novagen) plasmid DNA were digested with *NcoI* and *XhoI* and then ligated to generate pET28a-GGT construct. The construct substitutes the N-terminal signal peptide with *E. coli* specific equivalent and appends a C-terminal hexahistidine tag.

The construct was transformed into *E. coli* expression strain BL21 (Stratagene). For protein production, the cells were grown in LB medium supplemented with 30 *μ*g/mL kanamycin. When OD_600_ reached 0.5, the culture was induced by adding isopropyl-b-D-thiogalactopyranoside (IPTG) to a final concentration of 1 mM. Thereafter, the incubation was continued at 16°C overnight. The cells were pelleted by centrifugation at 6000 r.p.m. and stored at −20°C till further use.

### 2.3. Purification of GGT

IPTG induced cell pellet was suspended in 20 mM sodium phosphate buffer pH 7.0 and 500 mM NaCl (buffer A) containing 20 mM imidazole. The cell suspension was homogenised by two cycles of extrusion through French press at 1500 psi. The homogenate was clarified by centrifugation at 15000 r.p.m. for 1 hr and filtered through 0.45 *μ* membrane. The filtrate was loaded onto 1 mL HisTrap column (Amersham Biosciences) containing precharged high performance nickel sepharose, preequilibrated with buffer A and washed with the same buffer till OD_280_ of the effluent reached the baseline. The bound protein was eluted with a linear gradient of 20–500 mM imidazole contained in the binding buffer. The fractions corresponding to the single major peak were pooled and loaded onto Hi-Load Superdex S200 (Amersham Biosciences) column preequilibrated with 100 mM tris HCl pH 7.5 and 100 mM NaCl. Pure GGT eluted as a single prominent peak and was confirmed by activity test and SDS-PAGE analysis. The protein was concentrated to 10 mg/mL over a 10 kDa cutoff membrane (Pall) and preserved at −20°C.

### 2.4. Enzyme Assay

The enzyme was assayed by modified method of Suzuki et al. [16]. Briefly, 0.4 *μ*g of enzyme was incubated at 30°C for 5 mins with the ammonium salt of glutamyl-(3-carboxyl)-4-nitroaniline in 100 mM buffer in a final volume of 100 *μ*L. Buffers used were Tricine for pH 7.0–9.0 and *N*-cyclohexyl-3-aminopropanesulfonic acid (CAPS) for pH 10.0–11.0. The reaction was arrested with 900 *μ*L of 2 M acetic acid and the absorbance measured at 410 nm. Suitable blanks were used to correct for absorbance due to the substrate. One unit of enzyme was defined as µmoles of 3-carboxyl-4-nitroaniline formed in one minute. The assay was linear with respect to time of incubation.

### 2.5. Steady State Kinetics

Substrate saturation plots were prepared by measuring the reaction rates in a range of substrate concentrations. Concentrations were chosen to give a relatively even distribution of the data points. *K*_*m*_ and *V*_max⁡_ were determined by fitting the velocities to Michaelis-Menten equation by nonlinear regression method using Origin 6.1:

(2) $$\begin{array}{c} v=V_{\max}\frac{\left[S\right]}{K_{m}+\left[S\right]}. \end{array}$$

For measurements made at pH 10.0 and 11.0, the velocities were fitted by nonlinear regression to Hill's equation of the form:

(3) $$\begin{array}{c} v=V_{\max}\frac{{\left[S\right]}^{h}}{K_{0.5}+{\left[S\right]}^{h}}, \end{array}$$

where *v* is the initial velocity, *V*_max⁡_ is maximal velocity, *K*_*m*_ and *K*_0.5_ are the substrate concentrations for half maximal activity and *h* is the measure of cooperativity between *n* interacting sites.

### 2.6. Circular Dichroism (CD) Measurement

CD spectra were collected on a Jasco J-715 spectropolarimeter. Near and far UV CD spectra (200–250 nm) were collected with 0.1 and 1 mg/mL of the enzyme placed in a path length of 0.1 and 1 cm, respectively. The final spectrum was an average of 5 accumulations measured at a rate of 100 nm/min at 1 nm resolution.

### 2.7. Molecular Weight Estimation by Size Exclusion Chromatography

1x 100 cm Sephacryl S 200 was equilibrated with 150 mM NaCl in 50 mM Na-K phosphate buffer pH 7.0. The elution volume of blue dextran was considered as the void volume of the packed column. The column was calibrated by passing cytochrome C (12000 Da), carbonic anhydrase (24000 Da), bovine serum albumin (66000 Da), alcohol dehydrogenase (15000 Da), and beta-amylase (200000 Da). A calibration curve was constructed by plotting logarithm of the molecular weight of the individual marker against the ratio of the void (vo) and respective elution volumes (ve). A sample of *B. subtilis* GGT was passed through the calibrated column and its ve/vo ratio was used to calculate the molecular weight from the calibration curve. The column was then equilibrated with 100 mM CAPS buffer pH 11.0 and 150 mM NaCl and used to estimate the molecular weight of *B. subtilis* GGT as before.

### 2.8. Reversibility Study

A sample of the enzyme was incubated in 50 mM CAPS buffer pH 11.0 and 150 mM NaCl for 48 hrs at 6°C. The sample was then cleaned by dialysis against 100 mM tris HCl buffer pH 7.5 and 100 mM NaCl.

### 2.9. Thermodynamic Analysis

The value of *k*_cat_ for the respective reaction was determined at 20, 30, 40, and 50°C. Arrhenius plot was used to calculate the activation energy. The data were fitted by linear regression to the equation:

(4) $$\begin{array}{c} \ln k_{\text{cat}}=-\frac{E_{a}}{RT}+\ln A, \end{array}$$

where *E*_*a*_ is the activation energy, *R* is the gas constant, *T* is the absolute temperature, and *A* is the frequency of collision. The slop of this plot is equal to −*E*_*a*_/*R*.

Eyring's plot was used for the determination of enthalpy and entropy of activation. The data were fitted by linear regression to the equation:

(5) $$\begin{array}{c} \ln\frac{k_{\text{cat}}}{T}=\ln\frac{k_{B}}{h}+\frac{\Delta S^{‡}}{R}-\frac{\Delta H^{‡}}{RT}, \end{array}$$

where *h* is Planck's constant, *k*_*B*_ is Boltzmann's constant, *R* is gas constant, and *T* is absolute temperature. From the plot, Δ*H* ^‡^ was calculated from the slope ( = −Δ*H* ^‡^/*R*) and Δ*S* ^‡^ from the *y*-axis intercept. Free energy of activation (Δ*G* ^‡^) was calculated from the equation:

(6) $$\begin{array}{c} \Delta G^{‡}=\Delta H^{‡}-T\Delta S^{‡}. \end{array}$$

## 3. Results

### 3.1. Enzyme Preparation

*B. subtilis* GGT is expressed as single polypeptide precursor which undergoes posttranslational, intramolecular, and autocatalytic cleavage to produce a heterodimer [17]. The 2 polypeptide chains of the mature heterodimer weigh 45 and 22 KDa (Supplemental Figure 1 available online at http://dx.doi.org/10.1155/2014/216270). Expression of the active enzyme was temperature dependent. Induction of mid-log cultures at 30°C resulted in the formation of unprocessed precursor. Fully processed, heterodimeric enzyme was produced when the cultures were induced at 16°C.

### 3.2. *K*_*m*_ and pH Optimum

**Figure 1:**
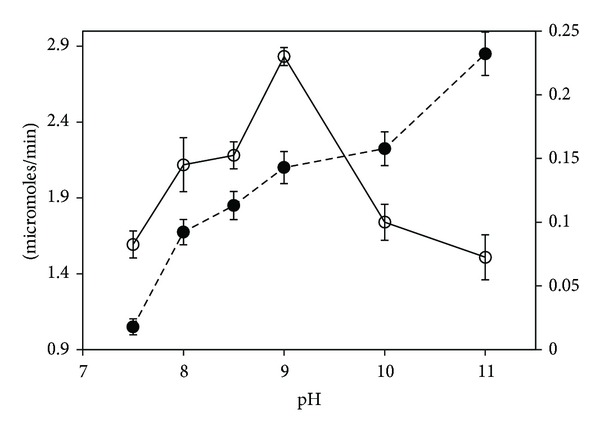
Effect of pH on *B. subtilis* GGT catalysed hydrolysis. Standard assay reactions were performed with either 2 mM (○) or 64 mM (●) *γ*-Glutamyl-(3-carboxyl)-4-nitroaniline.

The kinetics of GGT catalysed hydrolysis was studied with *γ*-glutamyl-(3-carboxyl)-4-nitroaniline as the substrate. The enzyme was active between pH 7.0 and 11.0. The shape of the pH titration curve and the optimum condition depended on the amount of substrate used in the assay. The optimum was at pH 9.0 when the assay used lower substrate concentration (2 mM). And, the optimum was at pH 11.0 when higher substrate concentration (64 mM) was used (Figure 1). *K*_*m*_ was found to be 25.9 ± 1.9, 16.7 ± 1.8, and 26.9 ± 2.7 mM at pH 7.5, 9.0, and 11.0, respectively. It was earlier reported to be 10.0 mM at pH 9.0 [18]. Thus, pH has negligible effect on the substrate affinity between pH 7.0 and 11.0. Among GGTs, the *K*_*m*_ of *B. subtilis* GGT is unusually high. The reported *K*_*m*_ values of other GGTs are mostly in micromolar concentrations: 68 *μ*M in *E. coli* GGT [16], 12 *μ*M in *H. pylori* GGT [19], 7 *μ*M in human GGT [20], 5 *μ*M in rat [21], 2 mM in rabbit GGT [22], and 1.6 mM in porcine GGT [23].

### 3.3. Kinetic Transformation

**Figure 2:**
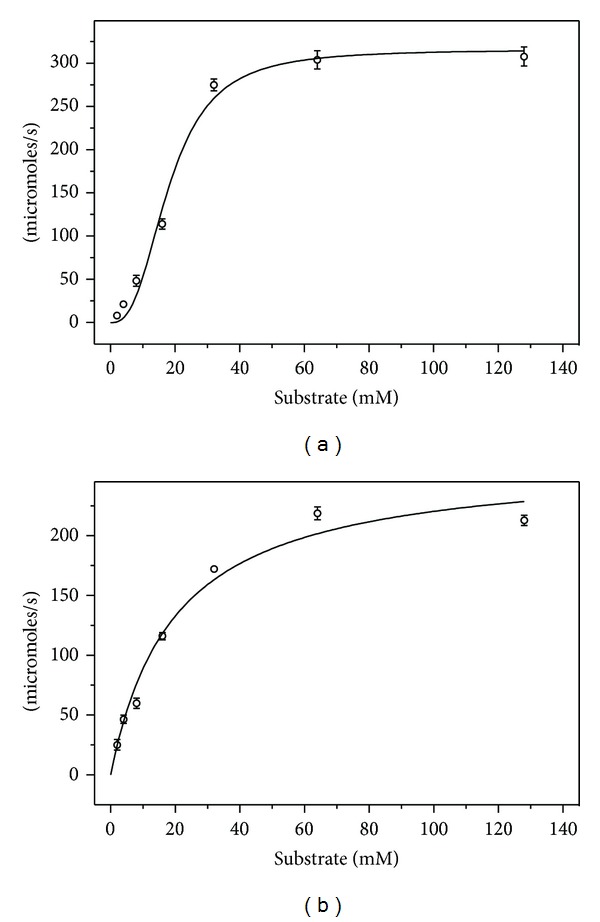
Effect of pH on the nature of saturation curve for *B. subtilis* GGT catalysed hydrolysis of *γ*-Glutamyl-(3-carboxyl)-4-nitroaniline. (a) and (b) represent substrate saturation curves at pH 9.0 and pH 11.0, respectively.

Between pH 7.0 and 9.0, the substrate saturation kinetics follows the standard Michaelis-Menten form producing a hyperbolic response. However, at pH 10.0 and 11.0, the substrate saturation curves were of sigmoid form (Figure 2). Standard deviation was higher (>10%) and adjusted coefficient of determination (*R* ^2^) was relatively lower when the data was fitted to hyperbolic curve. Better fit was obtained with Hill's equation (see supplementary Table 1 for details). The Hill's coefficient (*h*) was 1.6 ± 0.5 in pH 10.0 and 2.1 ± 0.4 at pH 11.0. Enzyme samples incubated at pH 11.0 for ~48 hours produced hyperbolic saturation curves when assayed at pH 9.0. This indicates that the kinetic transformation between hyperbolic and sigmoid forms is reversible and does not involve any permanent changes in the enzyme.

### 3.4. Effect of pH on Structure

The influence of pH on the structure of the enzyme was analysed to determine if any changes accompanied the kinetic transformation. Potential changes in the secondary and tertiary structures were analysed by CD spectroscopy. Absorbance in the far UV (200–250 nm) is mostly due to backbone amide bonds and therefore spectral changes in this region represent alterations in the secondary structure [24]. Aromatic residues (Trp, Tyr, and Phe) absorb most of the UV in the near region (250–300 nm); spectral changes in this region therefore are due to alterations in the tertiary structure [24]. CD spectra of *B. subtilis* GGT were collected in both far and near UV regions using samples present in pH 7.0 and 11.0 buffers. The spectra produced by samples in pH 7.0 and 11.0 were coincidental in both the UV regions (Supplemental Figure 2).

We then examined the effect of pH on the oligomeric state of the enzyme sample. The native molecular weight of the sample was analysed by gel-filtration method. The elution profiles in both pH 7.0 and 11.0 were coincidental and corresponded to a molecular weight of 66 KDa on the calibration curve (Supplemental Figure 3). Conservation of the molecular weight indicates that the sample does not undergo oligomerization at higher pH.

### 3.5. Thermodynamics of Hydrolysis across the pH Range

Thermal dependence of *k*_cat_ was determined in the range 20–50°C and used to prepare Arrhenius (Supplemental Figure 4(a)) and Eyring (Supplemental Figure 4(b)) plots. The experiments were performed in pH 7.5 and 11.0. The plots were linear in the experimental range. The thermodynamic parameters for the formation of the activated complex are given in Table 1. The activation enthalpies are positive as reactions involving bond breakage are endothermic. The negative value of Δ*S* ^‡^ indicates that the reactants must assume precise conformation and approach each other at precise angle to form the transition state and that the molecules are constrained in the activated state. The positive value for Δ*G* ^‡^ was expected as the formation of transition state is nonspontaneous. Thus, the *B. subtilis* GGT catalysed hydrolytic reactions in pH 7.5 and 11.0 are more or less thermodynamically similar, except for a small improvement in Δ*S* ^‡^at pH 11.0.

## 4. Discussion

The kinetics of GGT, particularly that of the mammalian homologues, have been extensively studied because of its physiological significance. Among microbial GGTs, the kinetics is mostly studied with the homologue from *E. coli*. Our detailed analysis of the enzymatic kinetics of *B. subtilis* GGT reveals interesting features that have so far been overlooked. Between pH 7.0 and 11.0, the *V*_max⁡_ of hydrolysis progressively increased producing maximum activity at pH 11.0. This was contrary to the previously reported pH profile wherein the hydrolysis followed a bell-shaped curve with the maxima at pH 9.0 [18]. Our analysis indicated that the reported optimum was an artifact as the pH profile was constructed not from *V*_max⁡_ values but from comparative assays at fixed substrate concentration. The reported assays employed merely 1 mM concentration of the substrate as the amount is the standard for most GGT assays, and, more importantly, the *K*_*m*_ of *B. subtilis* GGT was unknown at that time. The lowered rates at pH 10.0 and 11.0 relative to pH 9.0 under conditions of low substrate concentration were a consequence of kinetic transformation. At lower substrate concentrations, sigmoid kinetics typically produces slower rates than the corresponding hyperbolic form. We were able to recreate the bell-shaped curve by measuring the activities with 2 mM substrate and contrast it with measurements made with 64 mM substrate (Figure 1). The progressively increasing response at higher substrate concentration indicates the involvement of a critical acidic group that contributes to the catalysis in its deprotonated form.

The pH dependent transformation of the kinetics of substrate consumption from hyperbolic to sigmoid form is the most important observation in this study. From literature, we realised that a similar transitionary behaviour was observed previously in mammalian GGTs from human [25] and rabbit liver [22]. However, this aspect has not received much attention. The transition from hyperbolic to sigmoid form is sharp and occurs between pH 9.0 and 10.0. The kinetic data was analyzed using Hill's equation. This is a modification of Michaelis-Menten equation to account for multiple interacting binding sites. Hill's coefficient (*h*) represents cooperativity between multiple (*n*) binding sites. Hill's coefficient for hydrolysis in pH 10.0 and pH 11.0 was >1 indicating positive cooperativity between the interacting sites. The kinetic transformation is probably due to the involvement of a group with a p*K*_*a*_ of ~9.5, which upon deprotonation favours sigmoid kinetics. We need to explore if the critical deprotonated acidic group responsible for the maximal activity and the kinetic transformation are from the same amino acid residue. 

Sigmoid curve is generally a feature of enzymes with multiple substrate binding sites with dissimilar affinities. Binding of a substrate molecule at one of sites either improves or diminishes the binding affinity of the other sites. Multiple binding sites often arise due to oligomeric organization of the protein. Our size exclusion chromatography experiments under conditions of pH 7.5 and 11.0 gave same molecular weight, thus excluding oligomerization as the potential basis for sigmoid kinetics. Furthermore, CD spectroscopy data indicates the absence of any major structural changes. These results show that sigmoid kinetics arises due to nonconformational reasons.

GGT employs nucleophilic substitution mechanism of catalysis with the N-terminal threonine of the smaller subunit serving as the catalytic nucleophile [26]. The crystal structure of *B. subtilis* GGT in complex with glutamate has been determined [27]. The structure shows a glutamate residue bound at the catalytically competent active site. These facts show that there is only one active site in GGT including the *B. subtilis* homologue. The possibility of a second substrate molecule binding to the acceptor amine binding site is ruled out as sigmoid kinetics was evident in the transpeptidation reactions also (data to be published). The crystal structures of *B. subtilis* GGT have been determined both in the native and substrate conjugated forms [27]. The enzyme has a kidney shape with a groove on one side of the molecule. The active site, where the glutamate moiety of the substrate binds, occurs as a finger-shaped depression towards the middle of the groove. The region immediately outside of the active site does not show any large pockets that might bind to a glutamate residue. Therefore, it is unlikely that the sigmoid kinetics arises due to cooperative binding of multiple substrate molecules. Sigmoid kinetics due to monomeric enzyme with single substrate binding sites has been extensively studied in mammalian glucokinase. The sigmoid behaviour in glucokinase arises because of its occurrence in two interconvertible conformations with dissimilar affinities for the substrate [28]. Preequilibrium kinetics and nuclear magnetic resonance studies are necessary to elucidate the origins of sigmoid kinetics in *B. subtilis* GGT.

In conclusion, our study shows the drastic effect of pH on the hydrolytic kinetics of *B. subtilis* GGT. Our data rules out changes in substrate specificity, conformation, catalytic mechanism, and oligomeric state as the cause for the kinetic transformation.

## Supplementary Material

Supplementary Figure 1: PAGE gel showing purified *B. subtilis* GGT
Supplementary Figure 2: Circular dichroism spectra of *B. subtilis* GGT in near and far UV regions. Absorbance in pH 7.0 (*―*) and pH 11.0 (*―*) are shown.
Supplementary Figure 3: Calibration curve for the estimation of molecular weight by size exclusion chromatography. Elution point of *B. subtilis* GGT in pH 7.0 (*―*) and 11.0 (…) is marked.
Supplementary Figure 4: Analysis of thermal dependence of hydrolytic kinetics in pH 7.5 (○) and 11.0 (●). (A) Arrhenius Plots and (B) Eyring Plots.

## Figures and Tables

**Table 1:** Effect of pH on the thermodynamic parameters for the hydrolysis of *γ*-Glutamyl-(3-carboxyl)-4-nitroaniline.

pH	Activation energy (*E_a_*) KJ/mol	Activation enthalpy (Δ*H* ^‡^) KJ/mol	Activation entropy (Δ*S* ^‡^) J/mol K	Free energy of activation (Δ*G* ^‡^) KJ/mol
7.5	23.9	21.6	−147.7	65.6
11.0	27.9	25.4	−106.1	57.1
