# Supplementary material for: Effect of pH on the Hydrolytic Kinetics of Gamma-Glutamyl Transferase from Bacillus subtilis
Source: ScientificWorldJournal. 2014 Feb 24;2014:216270. doi: 10.1155/2014/216270 (PMC3956291; doi:10.1155/2014/216270)
Supplement: Supplementary file 1 — Supplementary Figure 1: PAGE gel showing purified B. subtilis GGT Supplementary Figure 2: Circular dichroism spectra of B. subtilis GGT in near and far UV regions. Absorbance in pH 7.0 (―) and pH 11.0 (―) are shown. Supplementary Figure 3: Calibration curve for the estimation of molecular weight by size exclusion chromatography. Elution point of B. subtilis GGT in pH 7.0 (―) and 11.0 (…) is marked. Supplementary Figure 4: Analysis of thermal dependence of hydrolytic kinetics in pH 7.5 (○) and 11.0 (●). (A) Arrhenius Plots and (B) Eyring Plots. [file 216270.f1.docx]

Supplementary Figure 1: PAGE gel showing purified *B. subtilis* GGT

Supplementary Figure 2: Circular dichroism spectra of *B. subtilis* GGT in near and far UV regions. Absorbance in pH 7.0 (―) and pH 11.0 (―) are shown.

Supplementary Figure 3: Calibration curve for the estimation of molecular weight by size exclusion chromatography. Elution point of *B. subtlis* GGT in pH 7.0 (_―_) and 11.0 (…) is marked.

Supplementary Figure 4: Analysis of thermal dependence of hydrolytic kinetics in pH 7.5 (○) and 11.0 (●). (A) Arrhenius Plots and (B) Eyring Plots.

Supplementary Figure 1

Supplementary Figure 2


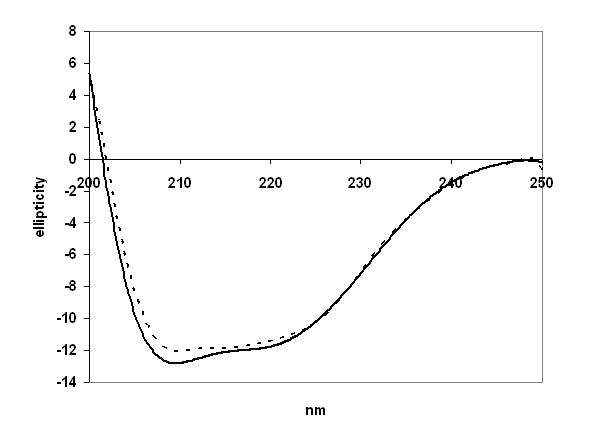
(A)


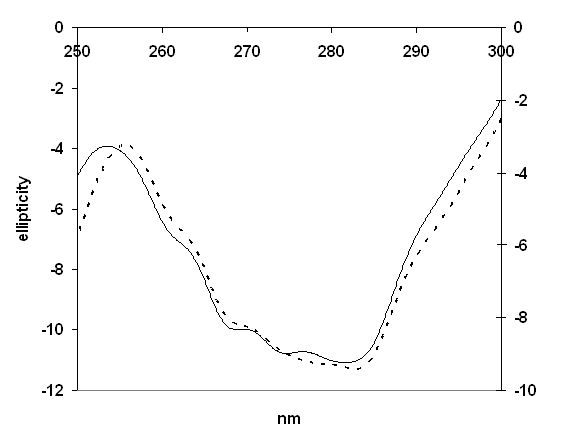
(B)

Supplementary Figure 3

Supplementary Figure 4

(A)

(B)
